# A Possible Role of FZD10 Delivering Exosomes Derived from Colon Cancers Cell Lines in Inducing Activation of Epithelial–Mesenchymal Transition in Normal Colon Epithelial Cell Line

**DOI:** 10.3390/ijms21186705

**Published:** 2020-09-13

**Authors:** Maria Principia Scavo, Federica Rizzi, Nicoletta Depalo, Elisabetta Fanizza, Chiara Ingrosso, Maria Lucia Curri, Gianluigi Giannelli

**Affiliations:** 1 Personalized Medicine Laboratory, National Institute of Gastroenterology “S. De Bellis” Research Hospital, Via Turi 27, Castellana Grotte, 70013 Bari, Italy; 2 Institute for Chemical-Physical Processes (IPCF)-CNR SS Bari, Via Orabona 4, 70125 Bari, Italy; federica.rizzi@uniba.it (F.R.); elisabetta.fanizza@uniba.it (E.F.); c.ingrosso@ba.ipcf.cnr.it (C.I.); marialucia.curri@uniba.it (M.L.C.); 3 Dipartimento di Chimica, Università degli Studi di Bari Aldo Moro, Via Orabona 4, 70125 Bari, Italy; 4 Scientific Direction, National Institute of Gastroenterology “S. De Bellis” Research Hospital, Via Turi 27, Castellana Grotte, 70013 Bari, Italy; gianluigi.giannelli@irccsdebellis.it

**Keywords:** tumor-derived exosomes, frizzled 10 protein, mesenchymal markers, epithelial–mesenchymal transition, colorectal cancer

## Abstract

Exosomes belong to the family of extracellular vesicles released by every type of cell both in normal and pathological conditions. Growing interest in studies indicates that extracellular vesicles, in particular, the fraction named exosomes containing lipids, proteins and nucleic acid, represent an efficient way to transfer functional cargoes between cells, thus combining all the other cell–cell interaction mechanisms known so far. Only a few decades ago, the involvement of exosomes in the carcinogenesis in different tissues was discovered, and very recently it was also observed how they carry and modulate the presence of Wnt pathway proteins, involved in the carcinogenesis of gastrointestinal tissues, such as Frizzled 10 protein (FZD10), a membrane receptor for Wnt. Here, we report the in vitro study on the capability of tumor-derived exosomes to induce neoplastic features in normal cells. Exosomes derived from two different colon cancer cell lines, namely the non-metastatic CaCo-2 and the metastatic SW620, were found to deliver, in both cases, FZD10, thus demonstrating the ability to reprogram normal colonic epithelial cell line (HCEC-1CT). Indeed, the acquisition of specific mesenchymal characteristics, such as migration capability and expression of FZD10 and markers of mesenchymal cells, was observed. The exosomes derived from the metastatic cell line, characterized by a level of FZD10 higher than the exosomes extracted from the non-metastatic cells, were also more efficient in stimulating EMT activation. The overall results suggest that FZD10, delivered by circulating tumor-derived exosomes, can play a relevant role in promoting the CRC carcinogenesis and propagation.

## 1. Introduction

Extracellular vesicles (EVs) are biological lipid-bilayer delimited particles secreted by every type of cells into the extracellular space. They can be broadly classified into three main classes, namely exosomes, microvesicles (MVs) and apoptotic bodies, based primarily on their size, biogenesis, cargo, function and release pathway during the cell life. Exosomes are vesicles with a size ranging from 50 to 150 nm, containing various bioactive molecules such as proteins (including oncoproteins, tumor suppressor proteins and transcriptional regulators), small RNAs and DNAs [1]. They are present in the bloodstream as efficient mediators of intercellular communication. Tumor-derived exosomes were recognized to deliver their oncogenic cargo from the primary tumor cells to healthy recipient target cells located in distant organs, with a significant implication in tumor progression and metastasis [2,3]. The hypothesis that metastases may originate from oncogenic macromolecules, circulating in plasma and derived from the primary tumor, which transfect susceptible cells, even in distant target organs, is known as “genometastasis” and was reported, for the first time, by Garcia-Olmo D. et al. [4]. The genometastatic hypothesis in human cells was recently confirmed by Abdouh S. et al. who proved the oncogenic transformation potential of cancer patient serum on human cells [5,6]. Exosomes were found to be involved not only in communication among cancer cells but also in reprogramming of mesenchymal stem cells (MSCs) to tumor sites. In this regard, Zakaria Y. A. E. et al. suggested that the neoplastic transformation of patient-derived adipose mesenchymal stem cells may result from their long-term exposure to tumor-derived oncogenic factors in prostate cancer (PC) patients. Furthermore, they indicated the active role of PC cell-derived exosomes as mediators of oncogenic transformation, genetic instability and mesenchymal–epithelial transition (MET) in patient-derived adipose stem cells [7]. Similarly, exosomes secreted by breast and ovarian cancer cells resulted able to convey the specific physical and functional features of tumor-supporting myofibroblasts to adipose tissue-derived mesenchymal stem cells [8].

Different proteins, such as vimentin, E-cadherin, N-cadherin, β-catenin and Slug/Snail, were found to be either upregulated or downregulated during the carcinogenesis, in particular during type 3 epithelial–mesenchymal transition (EMT), which is the process that converts epithelial cells into mesenchymal cells. In this transition, epithelial cells lose their characteristic cell polarity and cell–cell adhesion and gain migratory and invasive properties to become mesenchymal cells [9]. Generally, the downregulation of E-cadherin expression is observed in EMT mainly during tumor cell invasion [10]. This repression was related to the role of c-Myc oncogene in post-transcriptional mechanisms [11], cytoskeletal modification and cells adhesion that typically occur during tumorigenesis, as well as to the loss of epithelial markers and the gain of mesenchymal markers, such as vimentin [12]. During the carcinogenesis, the involved tissues were demonstrated to acquire rigidity compared to the normal tissues, due to an increase of cellular vimentin expression [13,14] and of cancer cell malignancy due to the re-organization of cell polarity [12,13]. In colorectal cancer (CRC), mutations in components actively involved in the Wnt/β-catenin signaling pathway induce deregulation of the c-Myc oncogene expression [15]. Recently, Frizzled 10 protein (FZD10), one of the membrane receptors involved in the Wnt pathway, was found to play a crucial role in CRC. Indeed, FZD10 resulted to be overexpressed not only in CRC tissues but also in exosomes derived from CRC patients and from CRC cell lines. Interestingly, an in vitro study suggested that FZD10 and FZD10-mRNA delivering exosomes may be potential messengers of disease reactivation even in quiescent cells. Moreover, FZD10 delivering exosomes are also thought to function as messengers of cellular transformation, with a concomitant possible active role in metastasis were supposed [16,17,18,19].

On the basis of these premises, this study investigated the possible role of FZD10 delivering exosomes, derived either from metastatic and non-metastatic CRC cell lines, to act as messengers able to trigger reprogramming of normal colon epithelial cells. To assess such a function of the FZD10 delivering exosomes, the acquisition of MCS-like features such as migration ability and expression of specific EMT involved proteins was evaluated.

## 2. Results

### 2.1. Exosomes Evaluation

**Figure 1:**
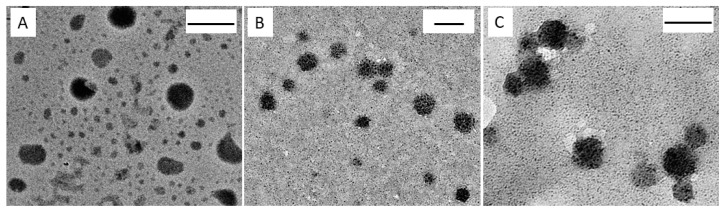
TEM micrographs obtained with positive staining of exosomes freshly extracted from culture medium of: HCEC-1CT cells (**A**); CaCo-2 cells (**B**); and SW620 cells (**C**). Scale bar, 100 nm.

Exosomes extracted from the culture medium of the untreated HCEC-1CT, SW620 and CaCo-2 cells were characterized in terms of morphology, size, size distribution and surface charge by TEM, DLS and ζ-potential investigation. A round shape can be observed for the exosome derived from culture medium of each cell line in TEM micrographs. Representative micrographs are reported in Figure 1, showing, in detail, spherical objects with size ranging from 30 to 100 nm.

DLS investigation of exosomes derived from HCEC-1CT, CaCo-2 and SW620 cells (Table 1) provides average hydrodynamic diameters lower than 150 nm and, therefore, compatible with the results of the TEM observation, considering that TEM analysis requires the preliminary samples deposition onto a grid and subsequently a drying step. Therefore, a shrinking of exosomes up to a certain extent is expected to occur, being exosomes aqueous vesicles derived from cell membranes and delimited by a phospholipid bilayer. Indeed, ζ-potential measurements (Table 1) revealed the presence of a negative charge on exosomes surface, ascribable to the negatively charged phosphate groups of the phospholipid bilayer.

**Figure 2:**
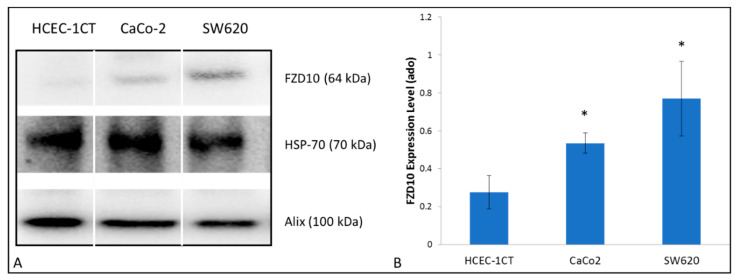
(**A**) Representative Western blotting of FZD10 and two exosomal protein markers (Hsp70 and ALIX); and (**B**) semi-quantitative estimation, by densitometry of protein bands, of relative FZD10 expression level in exosomes derived from the culture medium of HCEC-1CT, CaCo-2 and SW620. For each sample, the same total protein content was loaded (20 µg). Molecular mass markers are indicated on the right. For the semi-quantitative analysis, FZD10 bands are evaluated upon normalization with the corresponding housekeeping HSP-70 protein band, for each sample. (*) *p* < 0.001 versus HCEC-1CT cells.

Western blotting was performed on exosomes derived from the three different cell lines after extraction of their total protein content to investigate on the expression level of FZD10 (Figure 2). The semi-quantitative analysis proved an expression level of FZD10 in the tumor-derived exosomes significantly (*p* < 0.001 versus normal cells) higher than in the exosomes extracted from HCEC-1CT cell line. Moreover, the SW620-derived exosomes were found to present a level of FZD10 higher than that found in the CaCo-2-derived exosomes.

### 2.2. Exosomes Uptake by HCEC-1CT Cell Lines

**Figure 3:**
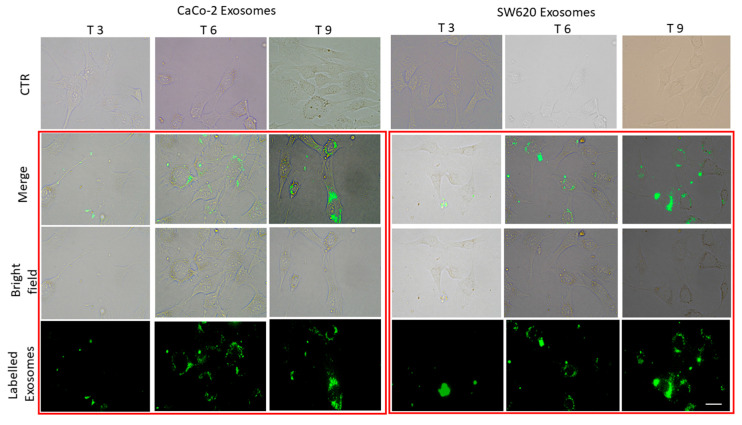
Confocal bright field and fluorescence micrographs of fixed HCEC-1CT cells. Time-dependent uptake of green fluorescent exosomes, freshly extracted exosomes from culture medium of CaCo-2 and SW 620 cells, in HCEC-1CT cells. Control (CTR) untreated cells. Micrographs of the cells after 3, 6 and 9 h of treatment with: CaCo-2-derived exosomes (A); and SW620-derived exosomes (B). Cells in the bright field images (Bright field), in green detection channel (labeled exosomes). Overlay of bright field and green fluorescence (Merge). Scale bar, 50 μm; magnification, 40×.

Normal epithelial cells HCEC-1CT cell lines were incubated with fluorescently labeled exosomes, derived from either non-metastatic CaCo-2 and metastatic SW 620 cells, at the final concentration of 100 μg (in terms of total protein content of exosomes)/100,000 cells, in order to monitor the cell uptake of exosomes proteins, at increasing incubation time (3, 6 and 9 h), by using confocal microscopy (Figure 3). After 3 h of incubation, the green fluorescent exosomes appeared localized on the surface of the cells, while their internalization was observed after 6 h. After a 9-h treatment, a perinuclear localization of fluorescent exosomes, in correspondence of the endoplasmic reticulum, was noticed. The time dependent cellular uptake for exosomes derived from the two different cancer cell lines, CaCo-2 and SW620 cells, was characterized by similar trend, as a complete internalization was observed after 6-h incubation.

### 2.3. Effect of Treatment with Exosomes on HCEC-1CT Migration

**Figure 4:**
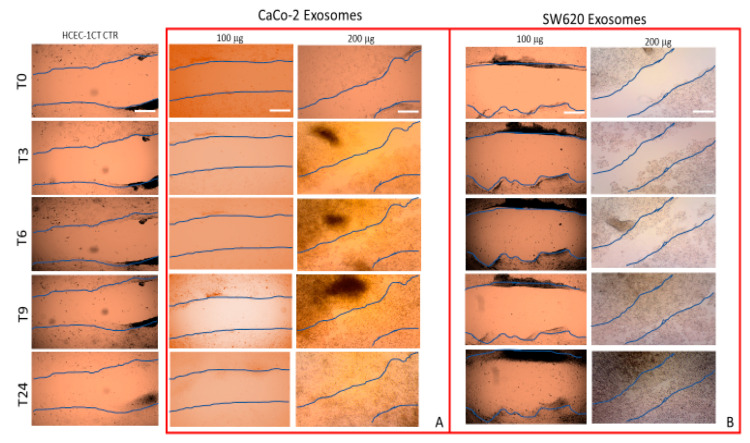
Qualitative analysis of collective HCEC-1CT cell migration by in vitro scratch assay. Representative micrographs of scratch-wound closure of cells treated with exosomes derived from the culture medium of CaCo-2 (**A**) or SW620 (**B**) cells at exosomes/cells concentration of 100 μg/100,000 and 200 μg/100,000 cells for 0, 3, 6, 9 and 24 h. CTR, untreated cells. Blue lines represent the edges of the scratched areas. Scal bar, 50 μm. Magnification, 10×.

In vitro scratch assay was performed to qualitatively evaluate the effect on the cells of the incubation with the exosomes, derived from the culture medium of the two cancers cell lines, CaCo-2 and SW620 cells, on the motility of normal epithelial HCEC-1CT cells, at different time points (Figure 4). A mechanical scratch (marked in blue) was made on semi confluent cell monolayers, and, subsequently, the HCEC-1CT cells were treated with exogenous exosomes at different exosomes/cells concentrations, namely 100 μg/100,000 cells or 200 μg/100,000 cells for 3, 6, 9 and 24 h. The first investigated time point, T0, is defined as the time when the exosomes were added to the cultures of the HCEC-1CT cells. Figure 4 shows that, when HCEC-1CT cells were incubated with exosome derived from the culture medium of CaCo-2 or SW620 cells, at exosomes/cells concentration of 200 μg/100,000 cells, the extent of the scratch wound appeared significantly reduced already within 6 h, if compared to corresponding control cells. A complete closure of the scratched area was observed after 24 and 9 h, for HCEC-1CT cells treated with exosomes, extracted from CaCo-2 and SW620 cells, respectively. However, in the case of the scratch healing at 24 h, a partial covering effect due to cell proliferation cannot be excluded, being doubling time equal to 18–24 h for HCEC-1CT cells.

No appreciable evidence of scratch closure over time was detected when HCEC-1CT cells were treated with exosomes isolated from the two different cancer cell lines at 100 μg/100,000 exosomes/cells concentration, compared to the corresponding untreated cells. Therefore, the overall results indicate that a sufficiently high exosomes/cells concentration is necessary to induce enhanced proliferation and an evident collective migration ability in normal epithelial cells, within 24 h.

### 2.4. Effect of Treatment with Exosomes on Deregulation of C-Myc, Vimentin and Other Proteins Involved in EMT on HCEC-1CT Cell Line

**Figure 5:**
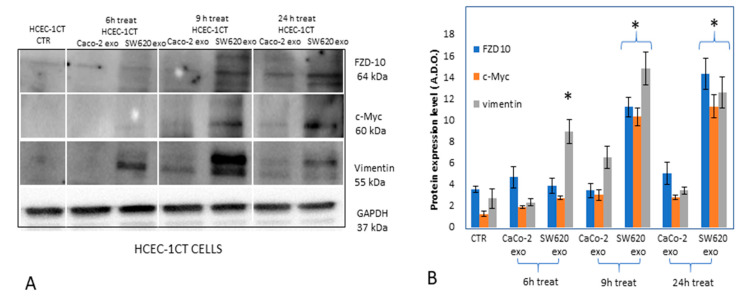
Representative Western blotting of FZD10, c-Myc, vimentin and GAPDH housekeeping protein (**A**) and semi-quantitative estimation, by densitometry analysis of protein bands, of relative FZD10, c-Myc and vimentin expression level (**B**) in HCEC-1CT cells, before and after treatment with exosomes derived from CaCo-2 (CaCo-2 Exo) or SW620 (SW620 Exo) cell lines for 6, 9 and 24 h. Control cells, untreated cells maintained in culture medium for 24 h before protein extraction. For each sample, the same total protein content was loaded (20 µg). Molecular mass markers are indicated on the right. For the semi-quantitative analysis, FZD10, c-Myc and vimentin bands are evaluated upon normalization with the corresponding housekeeping GAPDH protein band, for each sample. (*) *p* < 0.001 versus control.

Qualitative and semi-quantitative Western blotting analysis was carried out to evaluate the expression level of two EMT protein markers, c-Myc and vimentin, as well as of FZD10 in HCEC-1CT cells, before and after their treatment with exosomes, extracted by the culture medium of CaCo-2 or SW620 cells, at exosomes/cells concentration of 200 μg/100,000, for 6, 9 and 24 h (Figure 4). The exposure of HCEC-1CT cells to exosomes derived from metastatic SW620 was found to induce, in the samples investigated at 9 and 24 h of treatment, a statistically significant (*p* < 0.001 versus control cells) increase of the expression levels of c-Myc and FZD10 (Figure 5). A significant (*p* < 0.001 versus control cells) increase in the expression of vimentin was achieved already after 6 h of incubation. The HCEC-1CT cells treated with exosomes derived from non-metastatic CaCo-2 cells, did not show any statistically significant difference in the expression level either of vimentin, FZD10 or c-Myc.

**Figure 6:**
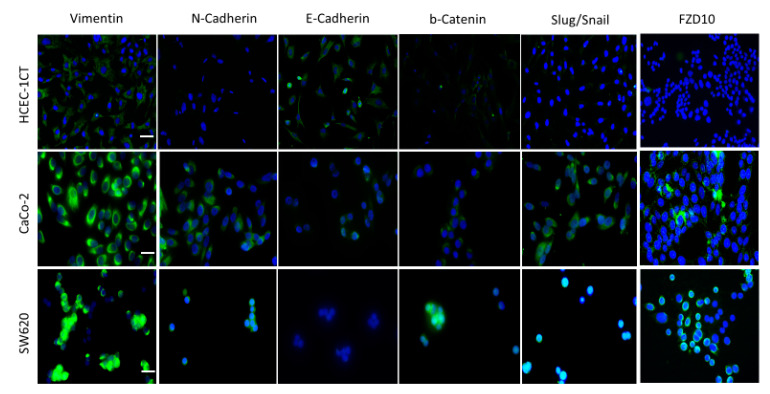
Detection of vimentin, N-cadherin, E-cadherin, β-catenin, Slug/Snail and FZD10 by immunofluorescence confocal microscopy in fixed HCEC-1CT, CaCo-2 and SW620 cells. Overlay of blue stained nuclei and green labeled proteins is shown for each cell line. Scale bar, 50 μm; Magnification, 40×.

Immunofluorescence imaging was performed to investigate possible modifications occurring in the expression of EMT protein markers, namely vimentin, N-cadherin, E-cadherin, β-catenin and Slug/Snail, as well as of FZD10 when HCEC-1CT cells were treated with exosomes derived from CaCo-2 or SW620 cell medium culture, with respect to the untreated cells. Therefore, as a first step, the detection of these proteins in untreated normal human colon epithelial (HCEC-1CT cells) and CRC cell lines (CaCo-2 or SW620 cells) was performed by immunofluorescence confocal microscopy (Figure 6).

**Figure 7:**
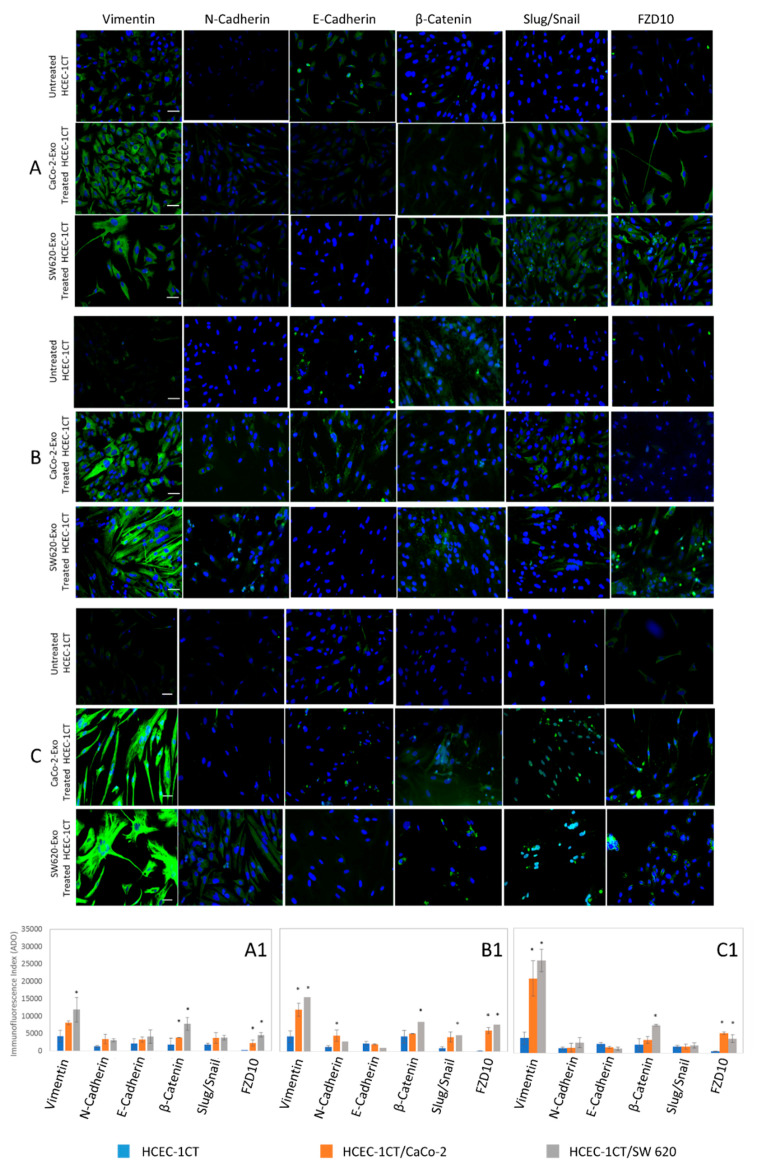
Detection of vimentin, N-cadherin, E-cadherin, β-catenin, Slug/Snail and FZD10 by immunofluorescence confocal microscopy in fixed untreated and exosomes treated HCEC-1CT cells over time. Confocal microscopy images and immunofluorescence data expressed by mean intensity index of HCEC-1CT cells, before and after treatment with the exosomes deriving from the CaCo-2 and the SW620 culture medium, for three days (**A**,**A1**); seven days (**B**,**B1**); and twelve days (**C**,**C1**). Overlay of blue stained nuclei and green labeled proteins was shown for each cell line. Scale bar, 50 μm; Magnification, 40×. * *p* < 0.001.

Confocal microscopy images of untreated HCEC-1CT cells (Figure 6) and the corresponding immunofluorescence by mean intensity index of vimentin, N-cadherin, E-cadherin, β-catenin, Slug/Snail and FZD10 (Figure 7, blue bars) indicated the presence of very low expression level of vimentin, N-cadherin, β-catenin, Slug/Snail and FZD10. In particular, the lowest expression was recorded for FZD10. Conversely, the expression of the E-cadherin, which is an epithelial and not mesenchymal marker, was clearly observed in the untreated HCEC-1CT cells, while it resulted less detectable in both the cancer cells. The confocal microscopy analysis highlighted the relevant expression of the FZD10 and all EMT marker proteins, except for E-cadherin, in both the colon cancer cell lines (Figure 6 and Figure 7, blue bars). The confocal microscopy images and immunofluorescence data expressed by mean intensity index of vimentin, N-cadherin, E-cadherin, β-catenin, Slug/Snail and FZD10 in HCEC-1CT cells, before and after their treatment with exosomes derived from both the cancer cell lines, at 3, 7 and 12 days, are reported in Figure 7A–C.

The immunofluorescence analysis shows a significant increase of fluorescence intensity index (*p* < 0.001 versus control cells) of vimentin and FZD10 upon exposure of HCEC-1CT cells to exosomes derived from both the two cancer cell lines, at each investigated incubation time. Expression levels of β-catenin and Slug/Snail were found to be statistically (*p* < 0.001 versus control cells) higher than in the untreated normal cells, at each tested treatment time and at seven days for β-catenin and Slug/Snail, only in the case of HCEC-1CT cells incubated with metastatic SW620-derived exosomes. A different localization of these two proteins was detected during the treatment of the cells. Condensation and translocation of β-catenin from the cytoplasm to the nucleus after three days of treatment of HCEC-1CT cells with SW620-derived exosomes was detected. Similarly, Slug/Snail was found to be localized in the cytoplasm and then in the nucleus when normal cells were treated with SW620-derived exosomes for 7 and 12 days, respectively.

## 3. Discussion

Exosomes, and generally all EVs, may act as vectors for delivery of specific biological information and as mediators of intercellular signaling within multicellular organisms in both health and pathological conditions. One of the mechanisms proposed to define the interaction between exosomes and plasma membrane of recipient cells is based on the concept of “vesicle–cell fusion”, a process that allows the vesicles to merge with the plasma membrane of a recipient cell. Different studies demonstrated the fusion of the exosomes’ membrane derived from cancer cells with recipient cells by using the fluorescent lipid mixing assay [20]. In this study, two different CRC cell lines, namely non-metastatic CaCo-2 cells and metastatic SW620 cells, were selected, and the corresponding exosomes were extracted from their cell culture medium to investigate possible exosomes mediated modifications that could be induced on normal colon epithelial cells (HCEC-1CT) in terms of migration ability and expression of FZD10, c-Myc and EMT markers. Firstly, the presence of FZD10 in the exosomes extracted from the three different cell lines was demonstrated and its expression level was quantitatively evaluated. The tumor-derived exosomes were characterized by significantly enhanced expression level of FZD10 compared to the exosomes derived from the normal cells. Interestingly, the exosomes isolated from the metastatic SW620 cells were found to express the highest level of the investigated protein. Such evidence confirms the observation of the presence of FZD10 in SW620-derived exosomes, already reported in a previous in vitro study [21]. Furthermore, FZD10 expression level was compared for normal and non-metastatic CRC cells. The ability of exosomes to be internalized by HCEC-1CT cells was investigated by labeling them with a green fluorescent tag. A complete cell uptake of the tumor-derived exosomes was observed after 6-h incubation of HCEC-1CT cells with exosomes derived from both the two CRC cell lines. The exosome uptake process can reasonably occur by vesicle–cell fusion mechanism and, accordingly, ends up in the release of their cargo within the normal cells, (Figure 3).

Western blotting analysis was performed to evaluate whether the normal HCEC-1CT cells exposure to the exosomes can stimulate their transformation, by inducing deregulation in the expression of vimentin and c-Myc, the two main proteins involved in the early stage of EMT, along with FZD10.

Their semi-quantitative analysis revealed that only the exosomes derived from metastatic SW620 induced a statistically significant over-expression of c-Myc, vimentin and FZD10 in HCEC-1CT cells within 24 h. Vimentin, one of the proteins expressed during the cell transition, plays a crucial role in embryonic development, wound healing, inflammation and regeneration, as well as in the oncogenic transformation and dissemination of a malignant tumor [22]. Therefore, vimentin is a well-established canonical marker of EMT reprogramming, associated with the acquisition of a migratory and invasive tumor cell phenotype [23], with an early overexpression during the transition.

The c-Myc oncogene is involved in the control of several normal cellular functions including growth, proliferation, migration, differentiation, angiogenesis and regulation of apoptosis mechanisms. The active role of overexpressed c-Myc as promoter of EMT and aggressive carcinoma has widely been demonstrated [24,25,26,27,28]. Aberrant expression of c-Myc was achieved in many human cancers and, it was found increased up to 70–80% in CRC [29].

In particular, during the carcinogenesis, dysregulation of Wnt/β-catenin signaling pathway is reported as a common event, that induces the nuclear translocation of β-catenin and upregulation of the expression of c-Myc, due to EMT activation [30,31]. A recent clinical study reported by Lee K. S. et al. evidenced the c-Myc and β-catenin over-expression in CRC patients [32]. FZD10 is a member of the frizzled gene family, which code for different proteins and function as receptors for the Wnt signaling proteins. Different studies suggested the active role of FZD10 during CRC progression, as its overexpression induced a consequent over-activity of Wnt/β-catenin [33]. The relevant involvement of the FZD10 in CRC was also proved by our in vitro study, which revealed the presence of FZD10 and FZD10-mRNA in exosomes extracted from culture medium of the metastatic CRC, gastric, hepatic and cholangial cancer cell lines, namely HGC-27, SW-620, N-87 and HUCCT-1 cells. A substantial reduction in FZD10 and FZD10-mRNA level in FZD10-mRNA silenced cells and in their corresponding exosomes was observed, and a significant decrease in viability of the silenced cells compared to their respective controls was demonstrated. The restoration of the cell viability, as well as of the FZD10 and FZD10-mRNA level, was promoted by incubation of silenced cells with the exosomes isolated by culture medium of the same untreated cells [21]. Remarkably, our clinical study established an active role for FZD10, delivered in small extracellular vesicles, as a potential biomarker for the early diagnosis of CRC and gastric cancer and for monitoring the treatment response [19].

Here, we found that the c-Myc oncogene, whose regulation is correlated to the canonical cascade of Wnt, was upregulated in normal cells, along with FZD10 and vimentin, upon stimulation by the exosomes derived from the metastatic SW620 cells, which were demonstrated to present a FZD10 level higher than that found in the exosomes isolated from the normal and non-metastatic cells. Therefore, the over-expression of c-Myc, vimentin and FZD10 induced on HCEC-1CT cells within 24 h by stimulation with exosomes derived from metastatic SW620 provides a clear indication of the activation of EMT in normal cells. Interestingly, an essential role of FZD10, that is an active receptor involved in the Wnt signal and delivered by exosomes, in the triggering the activation of the entire tumor cascade in normal cells, can be reasonably expected. Such an assumption is supported by the results reported by C. Gong et al. concerning the involvement of the FZD10 in the spread of the metastasizing signal, in breast cancer [34].

After assessing the early activation of c-Myc protein, vimentin and FZD10 during treatment with tumor-derived exosomes, the expression of other proteins involved in EMT, such as N-cadherin, E-cadherin, b-catenin and Slug/Snail, combined with vimentin and FZD10, was evaluated by immunofluorescence analysis at long exposure times, namely at 3, 7 and 12 days.

Statistically, a significant increase of vimentin and FZD10 expression was recorded when HCEC-1CT cells were incubated with exosomes isolated by either the two cancer cell lines, at each investigated incubation time. EMT is also governed by several signaling pathways that exhibit complex interactions with vimentin, such as snail and slug over-expression and migration of the proteins from cytoplasm into the nucleus, with a consequent increase of malignancy [35,36]. Snail and Slug are EMT inducing transcription factors, having a distribution that was found to depend on phosphorylation of a Ser-rich sequence adjacent to a nuclear export sequence (NES). D. Domínguez et al. demonstrated that the modification of such a sequence allows the nuclear export of the protein, and that phosphorylation and subcellular distribution of Snail are controlled by cell attachment to the extracellular matrix. These findings established the existence in tumor cells of an effective and fine-tuned non-transcriptional mechanism of Snail activity regulation dependent on the extracellular environment [37]. In our experiments, the expression of Slug/Snail increased over time in a statistically significant manner, when compared to the control cells, only when HCEC-1CT were treated with exosomes derived from metastatic SW620. Complete migration into the nucleus was evident after 12 days. Higher expression level of β-catenin was also achieved over time when HCEC-1CT were exposed to exosomes derived from metastatic SW620, and its localization into the cell nuclei was observed after three-day incubation. Aberrant Wnt signaling is a prominent feature of several cancers, especially CRCs, which was found in 90% of cases to exhibit overexpression of β-catenin [38,39].

The overall outcome of this study highlights that CRC-derived exosomes can be internalized by normal colon epithelial cells, and then release their cargo there in and induce cell reprogramming. The exosomes derived from the metastatic cell line, with a higher FZD10 level, were found to represent more efficient mediators of cell–cell communication, compared to exosomes extracted from the non-metastatic cells. They were able to trigger in normal cells the acquisition of an enhanced motility, which is an established mesenchymal feature, and, especially, the stimulation of EMT activation in the normal cell lines. Indeed, exposure of the normal cells to SW620-derived exosomes induced the over-expression of FZD10 along with specific EMT involved proteins.

Here, original experimental evidence indicates that FZD10 delivering exosomes may function as messengers of cellular modifications and metastasis process in CRC, although further investigation is needed to fully elucidate the process. The results of the performed investigation suggests that FZD10, a membrane receptor mediating the activation of the canonical pathway of Wnt and delivered by circulating tumor-derived exosomes, can represent a relevant player in promoting the CRC propagation, with autocrine and paracrine action and also above all in the metastatic sites, in the context of colorectal carcinogenesis.

## 4. Materials and Methods

### 4.1. Cell Culture

CaCo-2 human colorectal adenocarcinoma cells and metastatic SW-620 colon cancer cells were purchased from ATCC (LGC Standards S.r.l. Sesto San Giovanni, Milan, Italy). Human colon epithelial cell line HCEC-1CT was purchased from EverCyte (Evercyte GmbH. Vienna, Austria). All cell lines were cultivated accordingly to retailer protocols, using FBS depleted of exosomes, in all cultures.

### 4.2. Exosomes Extraction

The exosomes were extracted with the Total Exosome Isolation (from cell culture media) kit (Invitrogen, Carlsbad, CA, USA), according to the retailer protocols, in sterile conditions. After harvesting, the cell culture media was centrifuged at 2000× *g* for 30 min to remove cells and debris and, then, the supernatant was transferred to a new sterile tube. The required volume of the Total Exosome Isolation reagent was added and mixed with the medium by vortexing until a homogeneous solution was obtained and the tube was incubated in the fridge overnight. Successively, the mixture was centrifuged at 10,000× *g* for 1 h at 4 °C, the supernatant was discarded and the pellet-contained exosomes were resuspended in 200 μL of sterile water. The exosomes were then ready for their characterization, protein extraction or incubation with cells. The extracted exosomes were stored at −80 °C until proteins analysis was performed.

### 4.3. Transmission Electron Microscopy Investigation

Transmission Electron Microscopy (TEM) investigation was performed on exosomes by using a Jeol Jem-1011 microscope, working at an accelerating voltage of 100 kV. An Olympus Quemesa Camera (11 Mpx) was used to acquire the images. Staining of samples was performed according to the experimental procedure reported in Scavo M. P. et al. [21].

### 4.4. Dynamic Light Scattering (DLS) and ζ-Potential Investigation

The extracted exosomes were evaluated in terms of size distribution, hydrodynamic diameter, stability and corresponding polydispersity index (PDI) by using A Zetasizer Nano ZS (Malvern Instruments Ltd., Worcestershire, UK). The instrument operates with a 4 mW He-Ne laser as a light source (wavelength λ = 633 nm). A disposable folded capillary cell, DTS1070 (Malvern Instruments Ltd., Worcestershire, UK), was used, as already reported by Depalo et al. [40]. Three consecutive measurements were performed on each sample to obtain data reported as average value ± standard deviation.

### 4.5. Labeling of Exosomes

After their extraction from CaCo-2 and SW620 culture medium, the exosomes were fluorescently labeled by using the Green ExoGlow-Protein EV labeling kit (System Bioscience, Palo Alto, CA, USA), a fluorescent tag that specifically marks EVs protein cargo. The exosomes were treated according to the protocol form described by SBI. Briefly, exosomes were resuspended in 500 μL of sterile phosphate buffer (PBS 7.4 pH); then, the fluorescent tag was added and incubated with exosomes at 37 °C and shook for 20 min. After addition of 167 μL of ExoQuick-TC (System Bioscience, Palo Alto, CA, USA), the resulting mixture was further incubated overnight at 4 °C and, subsequently, centrifuged at 10,000 rpm for 10 min. The supernatant was carefully aspirated, while the exosomes were recovered as pellet and resuspended in a proper volume of PBS in order to obtain a concentration of total proteins suitable to proceed with the experiments. The total proteins content was evaluated by means of Bradford assay (Bio-Rad Hercules, CA, USA). The same procedure was used for untreated exosomes.

### 4.6. Exosomes Uptake

HCEC-1CT cells (100,000 cells/well) were seeded in a 6-well plate for uptake studies and when they reached 70% of confluence, 100 μg (in terms of total protein content) of labeled exosomes were added to the fresh medium in each well, and the uptake was monitored at fixed time points, namely at 0, 3, 6, 9 and 24 h (T_0_, T_3_, T_6_, T_9_ and T_24_), using the Eclipse Ti2 by Nikon confocal microscope (Nikon, Tokyo, Japan). The images were acquired by using a Kr-Ar and Ar lasers for the observation of labeled exosomes (488 nm) or bright field for visualization of the cells (40× magnification).

### 4.7. Migration Assay

For the migration assay, the scratch test was performed. Briefly, HCEC-1CT cells were seeded into 6-well plate (100,000 cells/well) and cultured by using the ColoUp medium (Evercyte GmbH. Vienna Austria) conditioned with exosomes derived from CaCo-2 or SW620 at two different protein concentrations (100 or 200 μg of total exosomes proteins for 100,000 cells) and supplemented with 10% of exosomes-depleted FBS (Evercyte GmbH. Vienna Austria), sodium pyruvate, 4.5 g/L glucose, 4 mM L-Glutamine and 5 mL Pen-Strep (Gibco, Waltham, MA, USA). Cells were grown until reaching semi-confluence, in a humidified incubator at 37 °C with an atmosphere containing 5% of CO_2_. Then, a scratch was made on the cell monolayer by using a p200 pipette tip. The plate was washed with sterile PBS and the medium replaced with ColoUp added with 5% of exosome-depleted FBS, and with exosomes derived from CaCo-2 or SW620 cell medium, at the two different reported concentrations.

In the case of untreated cells (control), only exosome-depleted cell cultured medium was used. The cell migration was monitored, capturing at regular intervals (every three hours) micrographs of the same region of the cell monolayer at 10× magnification. The micrographs were acquired and analyzed by using the Eclipse Ti2 by Nikon confocal microscope in bright field.

### 4.8. Western Blotting Analysis

Western blotting analysis was performed for total proteins extracted from exosomes derived from CaCo-2 and SW620 and for HCEC-1CT cells, untreated or treated with exosomes derived from both cancer cell lines, as previously described [39]. Untreated cells maintained in culture medium for 24 h were used as control. The method used for proteins extraction, total proteins amount quantification and the immunoblotting, was reported in Scavo M. P. et al. [21]. Anti-FZD10 (1:400 Abcam, Cambridge, UK), anti Alix (1:1000 Cell signaling, Danvers, MA, USA), anti-HSP-70 (1:500 Cell signaling, Danvers, MA, USA), anti-GAPDH (1:1000 Abcam, Cambridge, UK), anti-C-Myc and anti-Vimentin (both 1:1000 Cell signaling, Danvers, MA, USA) were used as primary antibodies and the Western blotting membranes were incubated with each of them overnight. Then, the membranes were treated with the corresponding HRP-conjugated secondary antibodies [1:1000 Santa Cruz, Santa Cruz, CA, USA] by following the previously reported protocol [21]. The chemiluminescence signals from proteins were imaged after incubation by using an enhanced chemiluminescence kit (Bio-Rad, Hercules, CA, USA) by Chemidoc XRS+ (Bio-Rad, Hercules, CA, USA). The images were analyzed by using Image Lab 5.2.1 software. Each experiment was repeated three times.

### 4.9. Immunofluorescence Analysis

Immunofluorescence analysis was performed using HCEC-1CT cells seeded into sterile slide chambers at a density of 1 × 10^3^ cells/well per each well. After 24 h, the cells were treated with exosomes derived from CaCo-2 or SW620 culture medium, at a concentration in terms of total exosome proteins equal to 200 μg/μL, for 15 days with administration of exosomes every 48 h. Every three days, a slide chamber for HCEC-1CT cells treated with exosomes derived from CaCo-2 or SW620 conditioned medium was fixed with cold 96° ethanol prior to immunofluorescence analysis and for EMT markers detection. CaCo-2 cells and SW620 were also analyzed by immunofluorescence to evaluate the expression of the EMT markers and FZD10 in each investigated cell line. The experiments were repeated three times. All cells lines (HCEC-1CT, CaCo-2 and SW620) were seeded into sterile slide chambers (8 wells each) at a density of 1 × 10^3^ cells/well at 37 °C. The immunofluorescence analysis was performed to detect the EMT markers in cells cultured with exosomes derived from CaCo-2 and SW620, using a concentration of total exosomes proteins already used in the other experiment before (200 μg/100,000 cells). The analysis was performed by following the same protocol reported in a previous study [21], using the primary antibodies against vimentin (rabbit polyclonal from Cell Signaling, Danvers, MA, USA), E-cadherin (rabbit polyclonal from BD, Franklin Lakes, NJ, USA), N-cadherin (mouse monoclonal from abCam, Cambridge, UK), β-catenin (rabbit polyclonal from abCam, Cambridge, UK), Slug/Snail (rabbit polyclonal from abCam, Cambridge, UK) and FDZ10 (rabbit polyclonal from abCam, Cambridge, UK). The cells were then incubated with a specific conjugated secondary anti-rabbit or anti-mouse IgG Alexa 488 (Invitrogen) for 1 h and mounted using prolong gold antifade reagent containing 4′,6-diamidino-2-phenylindole, DAPI (Invitrogen, Waltham, MA, USA). Images were acquired and analyzed by means of Eclipse Ti2 by Nikon confocal microscope. Kr-Ar laser with 488 nm band-pass filter was used for imaging vimentin, E-cadherin, N-cadherin, β-catenin, Slug/Snail and FZD10 (green channel), while Ar laser with 358-nm band-pass filter was used for imaging the DAPI for nuclear counterstaining (blue channel). All images were at 20× magnification. The fluorescence intensity was measured by using an exposure time of 200 ms and 1× gain per each acquisition for all the investigated samples and was quantified by means of Image-j software, by evaluating the number of pixel/areas. Each experiment was repeated three times.

### 4.10. Statistical Analysis

The Sigmastat 3.1 software was used for statistical analysis. Statistical significance between the two groups was assessed using the Student’s t-test (unpaired), and multiple comparisons were performed by using a one-way analysis of variance. The Kruskal–Wallis test was applied when the hypothesis of equality among groups was rejected by the one-way analysis of variance. We considered the occurrence of statistically significant difference between the results for each cell line and those of the corresponding untreated cells for *p* < 0.001.

## Figures and Tables

**Table 1:** Exosomes characterization by DLS analisys.

Cell Line Type	D_H_ (nm)	PDI	ζ-Potential (mV)
**HCEC-1CT**	106 ± 11	0.38 ± 0.18	−32.4 ± 4.5
**CaCo-2**	124 ± 6	0.32 ± 0.13	−56.3 ± 1.2
**SW 620**	150 ± 5	0.35 ± 0.06	−48.2 ± 3.2

Intensity-average hydrodynamic diameter and corresponding polydispersity index (PDI) determined by DLS and ζ-Potential value of the exosomes extracted from culture medium of all investigated cell lines and suspended in water. Mean value ± SD are reported, *n* = 3.
